# Intravesical aminophylline instillation as an alternative for balloon dilatation prior to semi-rigid ureteroscopic management of distal ureteral stones

**DOI:** 10.1007/s00345-022-04039-7

**Published:** 2022-05-26

**Authors:** M. Shabayek, T. Osman, M. Wahb, M. Elmoazen, D. Osman, A. Saafan

**Affiliations:** Urology department, Ain Shams University Hospitals, Abassia, 11361 Cairo Egypt

**Keywords:** Ureteral stones, Distal ureteral stones, Aminophylline, Intraureteral pressure, Ureteroscopy, Balloon dilatation

## Abstract

**Purpose:**

In a randomized controlled trial, we evaluated the effect of intravesical aminophylline instillation (IVAI) on intraureteral pressure of lower ureter and its use as an alternative to balloon dilatation after failure of advancing semi-rigid ureteroscope through the ureteric orifice without endodilatation.

**Methods:**

Our study included 83 patients with juxta-vesical distal ureteral calculi requiring endodilatation after unsuccessfully introducing the semi-rigid ureteroscope through the ureteric orifice. Patients were randomized into two groups: group A (study group) included 41 patients, where IVAI was used to dilate the ureter and facilitate ureteroscopy (the intraureteral pressure was measured using a pressure transducer connected to an invasive pressure monitor before and 5 min after IVAI), whereas group B (control group) included 42 patients, where balloon dilatation was used prior to ureteroscopy. Perioperative surgical outcomes of ureteroscopy were evaluated in both groups.

**Results:**

A statistically significant decrease in mean intraureteral pressure of intravesical ureter was found after IVAI from 12.34 mmHg ± 1.94 before injection to 8.46 mmHg ± 1.94 after injection (*P* < 0.001). Ureteral injuries, postoperative pain and hematuria were statistically significantly less among the study group compared to the control group (*P* < 0.05). We did not find statistically significant differences in operative time, need for DJ ureteral stenting or stone-free rate between both groups and no perioperative side effects were associated with IVAI.

**Conclusion:**

In ureteroscopic management of distal ureteral stones, intravesical aminophylline instillation is safe, inexpensive and effective in reducing intraureteral pressure and achieves comparable outcomes to balloon dilatation with less ureteral injuries, postoperative pain and hematuria.

## Introduction

Semi-rigid ureteroscopy is the treatment of choice for distal ureteric stones, being a cost-effective method with low morbidity, short hospital stay and high stone-free rate [1]. Ureteral dilatation prior to ureteroscopy is frequently required and should be simple, efficient and with minimal iatrogenic trauma [2]. Local intravesical aminophylline instillation (IVAI) was reported to facilitate ureteroscopy and transureteral lithotripsy with reduced perioperative complications and increased stone-free rate [3].

We present the first clinical study to demonstrate the effect of IVAI on intraureteral pressure at the distal ureter and to evaluate its safety and efficacy as an alternative for balloon dilatation during ureteroscopy.

## Patients and methods

During the period from October 2018 to October 2020, 365 patients with juxta-vesical lower ureteric stone ≤ 2 cm were evaluated. All patients were initially given medical expulsive therapy (MET) and tamsulosin and re-evaluated after 2 weeks. Patients with ureteric stones ≥ 10 mm on non-contrast computer tomography for urinary tract (NC-CTUT) and/or moderate to severe hydroureteronephrosis or severe pain were also given MET and tamsulosin and scheduled for prompt ureteroscopy. Comprehensive preoperative evaluation included medical and surgical history, complete physical examination, preoperative laboratory investigations and NC-CTUT.

**Fig. 1:**
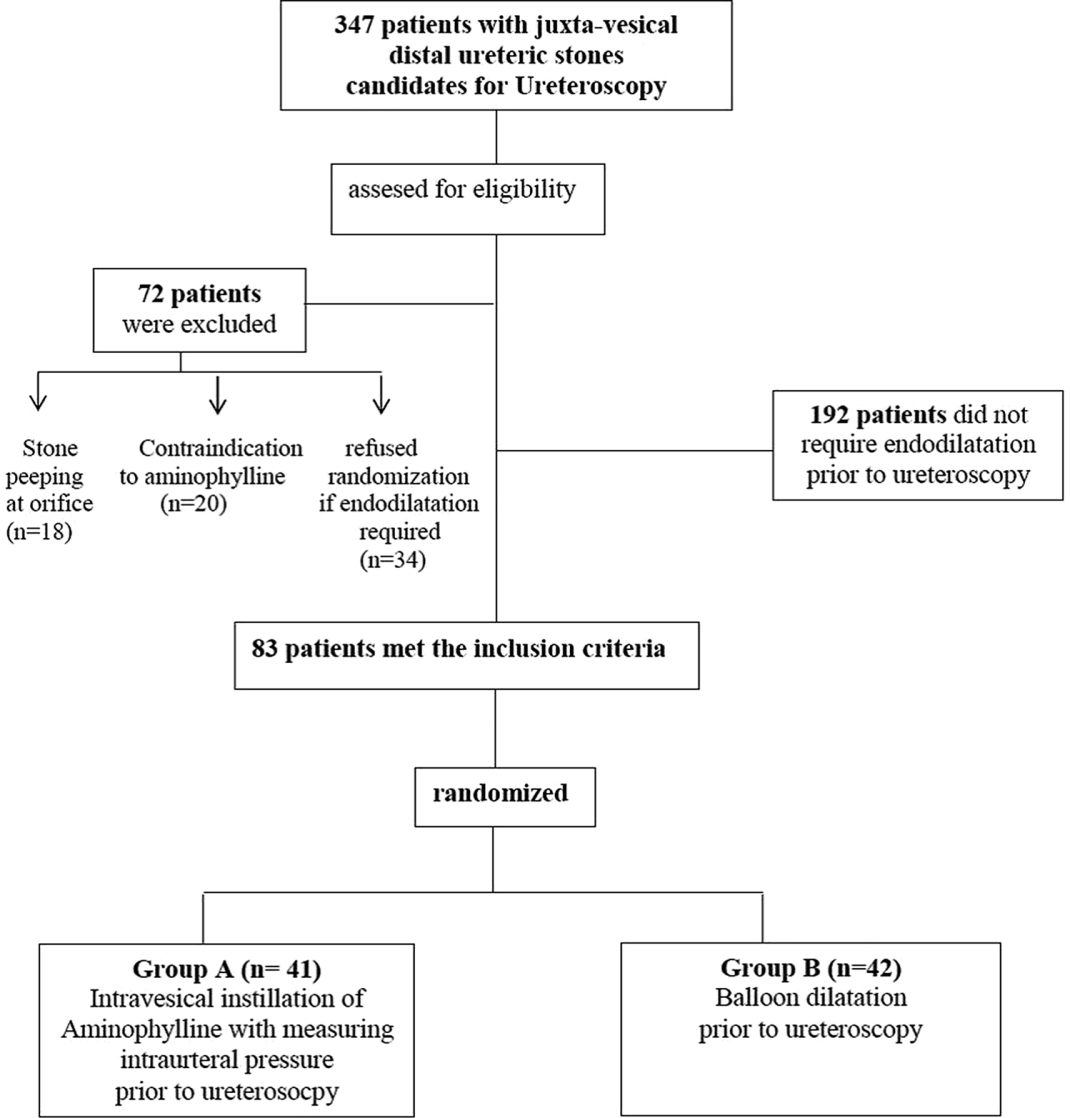
CONSORT chart of the clinical trial

We excluded patients with stones peeping at the ureteric orifice, having anatomic or functional renal disorders or any contraindication for aminophylline use; hypersensitivity to drug components, pregnancy or lactation (absolute contraindication); or presence of cardiologic, neurologic diseases, renal impairment or hepatic dysfunction (relative contraindication). Eighty-three patients were included in this randomized controlled trial (RCT) (Fig. 1).

In each case, direct semi-rigid ureteroscopy (using 8/9.5 fr. ureteroscope 27002L Karl Storz®, Germany) was initially attempted without endodilatation. On failure to advance the semi-rigid ureteroscope through the ureteric orifice, ureteroscopy was temporarily aborted and patients were randomly assigned to either IVAI followed by ureteroscopy (group A) or conventional ureteric balloon dilation before ureteroscopy (group B). Randomization was done through computer-generated list and sealed envelopes. In group A, intraureteral pressure was measured before and 5 min after IVAI.

All patients were operated under the same spinal anesthesia protocols and similar intravenous hydration. Patients were asked to fast overnight for 8 h and were given a third-generation cephalosporin as a prophylactic antibiotic at induction of anesthesia. Continuous intraoperative vital data, blood pressure, pulse and respiratory rate, were monitored by the attending anesthologist to detect any possible systemic adverse effect of aminophylline.

Group A (study group) included 41 patients, who had their intraureteral pressure measured before and 5 min after intravesical instillation of 150 ml of the prepared solution (normal saline with aminophylline 250 mg/10 mL). The intraureteral pressure was measured using a pressure transducer connected to invasive pressure monitor by a 6-Fr double-lumen Amecath® urodynamic catheter introduced through the ureteric orifice distal to the ureteric stone. A safety guide wire was inserted prior to ureteroscopy without further ureteral dilatation. Group B (control group) included 42 patients; where a safety guide wire was initially inserted under fluoroscopy, dilatation of the lower ureter was achieved by Uromax™ balloon catheter at 20 atm for 2 min. Thereafter, a semi-rigid ureteroscope was introduced alongside the guide wire. Gentle trials to extract stones < 10 mm by stone forceps were performed initially. Failed gentle extraction of stones < 10 mm, stones ≥ 10 mm and/or impacted stones were fragmented with pneumatic lithotripsy before extraction. Holmium laser lithotripsy was spared for upper ureteric stones and flexible ureteroscopy (more cost-effective). At the end of the procedure, either a double J (DJ) stent or ureteric catheters were applied according to ureteral wall status and the presence of residual stones.

We evaluated stone-free rate (SFR), defined as no residual stones or insignificant residual fragments < 4 mm as found on postoperative NC-CTUT. All patients were monitored for pain after resolution of the effect of spinal anesthesia with visual analog scales every 4 h. Postoperative pain was evaluated through the need for analgesics in the first 24 postoperative hours. Postoperative complications, fever, hematuria, urinary tract infection and pyelonephritis, as well as postoperative aminophylline toxicity (tachycardia, arrhythmias, hypertension, seizures, restlessness, insomnia) were evaluated. We also assessed the need for postoperative extracorporeal shockwave lithotripsy (SWL) or re-do ureteroscopy.

Patients were followed up 3 months after discharge to assess 90-day postoperative complications through comprehensive history, focused clinical examination, urinanalysis, pelvi-abdominal ultrasonography and/or NC-CTUT as indicated.

## Statistical analysis

Data were collected, coded and analyzed by the Statistical Package for Social Science (SPSS) version 23. Quantitative variables were presented as mean, standard deviations (± SD), while categorical variables were presented as numbers and percentages. The association of categorical variables was analyzed by Chi-square test and Fisher exact test (when the expected count in any cell was less than 5). The comparison between two independent groups with quantitative data and parametric distribution was done by independent *t* test***.**** P* value was considered significant if < 0.05 and highly significant if < 0.005.

## Results

The mean age of patients included in our study was 38.1 ± 8.8 years. Fifty-eight patients (69.9%) were males. Nineteen patients (22.9%) had recurrent stone formation and 16 patients (19.3%) had previous ureteroscopy. Both groups were matched with no statistically significant differences in preoperative characteristics (Table 1).

**Table 1:** Preoperative characteristics of patients included in our study

		Group A (received aminophylline) (*n* = 41)	Group B (balloon dilatation) (*n* = 42)	Test value	*P* value
Age	Mean ± SD	37.83 ± 8.95	38.36 ± 8.8	0.271	0.787
Sex	Male	30 (73.2%)	28 (66.7%)	0.417	0.518
	Female	11 (26.8%)	14 (33.3%)		
FH for urolithiasis	No	29 (70.7%)	33 (78.6%)	0.675	0.411
	Yes	12 (29.3%)	9 (21.4%)		
Stone recurrence	No	32 (78%)	32 (76.2%)	0.041	0.84
	Yes	9 (22%)	10 (23.8%)		
Stone size	Mean ± SD	10.49 ± 3.23	9.48 ± 3.51	1.367	0.176
	1 –4 mm	3 (7.3%)	4 (9.5%)	0.975	0.614
	5 – 9 mm	16 (39%)	20 (47.6%)		
	10 – 20 mm	22 (53.7%)	18 (42.9%)		
Previous URS	No	31 (75.6%)	36 (85.7%)	1.361	0.243
	Yes	10 (24.4%)	6 (14.3%)		

*FH* family history, *URS* ureteroscopy

Ureteral access was successful in all patients after IVAI in group A and after balloon dilatation in group B. There was a statistically significant (*P* < 0.001) decrease in intraureteral pressure after IVAI (8.46 mmHg ± 1.94) compared to pressure before injection (12.34 mmHg ± 1.94). Mean operative time was relatively longer, but not statistically significant in group A than in group B (*P* = 0.126). Gentle extraction of stones 5–9 mm without lithotripsy was successful in 62.5% (10/16) and 65% (13/20) in group A and group B, respectively. We found no statistically significant difference with regard to the need for lithotripsy between both groups (*P* = 0.406). Ureteral wall injuries were statistically significantly (*P* = 0.047) higher in group B (19%) compared to group A (4.9%). Balloon dilatation was associated with ureteral mucosal tear in 9.5%, submucosal false passage in 7.1% and ureteral perforation in 2.4% compared to ureteral mucosal tear in only 4.9% with IVAI. The need for intraoperative DJ stenting was not statistically significantly different between both groups (*P* = 0.335). No intraoperative cardiovascular manifestations (tachycardia, arrhythmias or hypertension) or seizures from IVAI were observed (Table 2).

**Table 2:** Intraoperative evaluation

			Group A (received aminophylline) (*n* = 41)	Group B (balloon dilatation) (*n* = 42)	Test value	*P* value
Operative time	Mean ± SD		38.1 ± 6.98	35.64 ± 7.34	1.545	0.126
Lithotripsy	No		13 (31.7%)	17 (40.5%)	0.691	0.406
	Stone size	1-4 mm	3 (7.3%)	4 (9.5%)		
		5-9 mm	10 (24.4%)	13 (31%)		
	Yes		28 (68.3%)	25 (59.5%)		
	Stone size	5–9 mm	6 (14.6%)	7 (16.7%)		
		10-20 mm	22 (53.7)	18 (42.9%)		
Ureteral injury	No		39 (95.1%)	34 (81%)	3.931	0.047*
	Yes		2 (4.9%)	8 (19%)		
Stenting after ureteroscopy	Ureteral catheter		33 (80.5%)	30 (71.4%)	0.931	0.335
	DJ stent		8 (19.5%)	12 (28.6%)		

*SD* standard deviation, *DJ* Double J

^*^Statistically significant

All patients were discharged 24 h after radiologic evaluation with NC-CTUT to assess SFR. Ureteric catheters were removed before discharge from hospital and DJ stents were removed after 2–3 weeks or after SWL if required.

Overall, SFR at first postoperative day was 88% (73/83). Stone-free rates were 87.8% (36/41) and 88.1% (37/42) in group A and group B, respectively, with no statistically significant difference in SFR between both groups (*P* = 0.968). Eight patients with residual stones underwent either SWL in 8.4% (7/83) or re-do ureteroscopy in 1.2% (1/83), whereas two patients with residual fragments 5–6 mm continued on medical treatment. Patients in group A had statistically significant less postoperative pain (*P* = 0.022) and required less analgesic (19.5% versus 42.9%). Postoperative hematuria was also statistically significant less in group A than in group B (*P* = 0.03). No postoperative IVAI associated toxicity was reported.

All patients were stone free at 3 months follow-up and we did not find statistically significant difference in 90-day postoperative urinary tract infection or pyelonephritis between both groups (Table 3).

**Table 3:** Postoperative outcomes

		Group A (received aminophylline) (n-41)	Group B (balloon dilatation) (n-42)	Test value	*P* value
SFR﻿	Free	36 (87.8%)	37 (88.1%)	0.002	0.968
	Residual	5 (12.2%)	5 (11.9%)		
Pain	No pain medication needed	33 (80.5%)	24 (57.1%)	5.256	0.022*
	Relieved by medication	8 (19.5%)	18 (42.9%)		
Hematuria	No	37 (90.2%)	30 (71.4%)	4.72	0.030
	Yes	4 (9.8%)	12 (28.6%)		
Management of residual stones	SWL	3 (66.7%)	4 (100.0%)	–	1.000
	Re-do URS	1 (33.3%)	0 (0.0%)		
90-days UTI	Yes	3 (7.3%)	4 (9.5%)	–	1.000
	No	38 (92.7%)	38 (91.5%)		
90-days Pyelonephritis	Yes	2 (4.9%)	3 (7.1%)	–	1000
	No	39 (95.1%)	39 (92.9%)		

*SFR* stone free rate, *SWL* extracorporeal shock wave lithotripsy, *URS* ureteroscopy, *UTI* urinary tract infection

*Statistically significant

## Discussion

Few studies evaluated upper tract pressure using different methods [4, 5]. Shafik used 3-Fr ureteric catheter to measure the ureteral pressure via a transducer output recorded on a rectilinear recorder [4]. Cai and colleagues measured the intraureteral pressure proximal to the stone after fragmentation during ureteroscopy via irrigation fluid through a ureteroscope connected to a pressure transducer recording the pressure on a pressure monitoring device [5]. The intraureteral pressures measured by Shafik [4] and Cai and colleagues [5] 7.7 mmHg and 10.2 mmHg, respectively, were lower than the mean intraureteral pressure in our study (12.2±1.85 mmHg).

Several animal studies evaluated the effect of different medications on the modulation of ureteral contractions, peristalsis and smooth muscle relaxation of the upper urinary tract [6–8]. However in humans, only Jung and colleagues [9] demonstrated a statistically significant reduction in mean pelvic pressure using isoproterenol compared to saline instillation during flexible ureterorenoscopy.

Aminophylline is an inexpensive drug that has been widely available for decades. It is rapidly absorbed and converted to theophylline. The possible mechanism of action of theophylline is inhibition of phosphodiesterase enzyme (PDE), increasing the intracellular cyclic adenosine monophosphate (cAMP) and cyclic guanosine monophosphate (cGMP) levels leading to smooth muscle relaxation. Additional anti-inflamatory effects of theophylline are through adenosine receptor antagonism, inhibiting the release of inflammatory mediators, histamine, leukotrienes and superoxide anions, as well as stimulating the release of anti-inlamamtory mediator interleukin 10 (IL-10) through inhibition of PDE. Theophylline also activates histone deacetylase downregulating the expression of inflammatory genes [10].

We demonstrated 3.88 mmHg mean reduction of intrautereal pressure at the distal ureter (12.34 mmHg and 8.46 mmHg before and after IVAI, respectively) and this decrease in intraureteral pressure could probably replace ureteral dilatation prior to ureteroscopic extraction and lithotripsy of distal ureteric stones. Decrease in ureteral pressure was associated with easy access through the ureteric orifice without endodilation, statistically significant less ureteric wall injuries, less hematuria and pain as well as comparable SFR.

The effect of local aminophylline instillation into the renal collecting system was studied by Green and colleagues, who demonstrated smooth muscle relaxation and facilitated intra-renal access to calyceal staghorn stones in ureteropelvic junction and/ or infundibular spasms [11].

A previous double-blinded RCT by Barzegarnezhad and colleagues [3] evaluated outcomes after IVAI compared to intravesical saline instillation in ureteroscopy for lower ureteric stones.

In our study, there was no statistically significant difference regarding SFR and operative time between cases which underwent IVAI or balloon dilatation; however, Barzegarnezhad and colleagues [3] demonstrated a statistically significantly higher SFR (95% versus 76.1% respectively) and shorter duration for lithotripsy (*P* = 0.01) in favor of IVAI compared to saline instillation.

Barzegarnezhad and colleagues [3] also reported highly statistically significant reduction in both intraoperative need for DJ insertion and postoperative SWL (P = 0.001) with IVAI compared to saline instillation. Although our study demonstrated statistically significant less ureteral injuries (P = 0.047), we did not find statistically significant difference regarding the need for DJ insertion or the need for postoperative SWL between both groups (*P* = 0.335 and *P* = 1 respectively).

A major limitation for systemic use of aminophylline is its adverse effects. Nausea, vomiting and headache are associated with PDE4 inhibition, whereas palpitation and cardiac arrhythmias are associated with inhibition of PDE3. Neurologic stimulation, gastroesophageal reflux and diuresis are due to adenosine antagonism. Several drugs, diseases and diet may influence theophylline clearance through modulation of cytochrome P450 enzyme, e.g., ciprofloxacin, erythromycin, cimetidine and allopurinol, as well as hepatic diseases, pneumonia, renal impairment and congestive heart failure [10].

In corcordance with the study of Barzegarnezhad and colleagues [3], we found no perioperative IVAI-related side effects.

Djaladat and colleagues [12] studied the effect of aminophylline on renal colic and reported that aminophylline can decrease renal pain and reduce the need for narcotics. Our study demonstrated statistically significant difference in both postoperative pain and hematuria after IVAI compared to balloon dilatation (*P* = 0.04 and *P* = 0.03, respectively).

A noticeable finding in our study was statistically significant reduction of ureteral wall injuries with IVAI compared to conventional balloon dilation. IVAI was also associated with less ureteral DJ stenting (19.5%) compared to balloon dilation (28.6%); however, this was not statistically significant.

To our knowledge, no previous clinical study evaluated the effect of IVAI on distal intraureteral pressure. Further studies should be encouraged to evaluate the best method for measuring intraureteral pressure and to study the effect of aminophylline and other pharmacologic agents to enhance the outcomes of ureteroscopic management of ureteric stones. The main limitation of our study is the small sample size. Large multicenter clinical trials are recommended to ascertain the use of IVAI as an alternative to balloon dilatation during extraction and lithotripsy of distal ureteric stones. Moreover, longer follow-up is needed to examine if the use of aminophylline solution for ureteral dilation (that causes minimal or no trauma to ureteral mucosa) instead of conventional balloon dilation may be associated with lower ureteric stricture rate or not.

## Conclusion

Intravesical aminophylline instillation is safe, inexpensive and effective in reducing intraureteral pressure at the lower ureter and can be used as an alternative to balloon dilatation prior to ureteroscopic management of distal ureteric stones.

## Data Availability

The datasets used and/or analyzed for the current study are available from the corresponding author on request.

## Abbreviations

Atm: Atmosphere
cAMP: Cyclic adenosine monophosphate
cGMP: Cyclic guanosine monophosphate
DJ: Double J-stent
Fr: French
IL-10: Interleukin 10
IVAI: Intravesical aminophylline instillation
KUB: X-ray for kidney, ureter and bladder
MET: Medical expulsive therapy
NC-CTUT: Non-contrast computer tomography for urinary tract
PDE: Phosphodiestrase enzyme
RCT: Randomized controlled trial
SD: Standard deviation
SFR: Stone free rate
SPSS: Statistical package for social science
SWL: Shock wave lithotripsy
